# Therapeutic potential of mesenchymal stem cell-derived exosomes in skeletal diseases

**DOI:** 10.3389/fmolb.2024.1268019

**Published:** 2024-06-06

**Authors:** Xiaobo Yang, Shaodian Zhang, Jinwei Lu, Xiaoling Chen, Tian Zheng, Rongxin He, Chenyi Ye, Jianbin Xu

**Affiliations:** ^1^ Department of Orthopedic Surgery, the Second Affiliated Hospital, Zhejiang University School of Medicine, Hangzhou, Zhejiang, China; ^2^ Orthopedics Research Institute of Zhejiang University, Hangzhou, Zhejiang, China; ^3^ Key Laboratory of Motor System Disease Research and Precision Therapy of Zhejiang Province, Hangzhou, Zhejiang, China; ^4^ Clinical Research Center of Motor System Disease of Zhejiang Province, Hangzhou, China; ^5^ Department of Plastic Surgery, The Fourth Affiliated Hospital, Zhejiang University School of Medicine, Yiwu, Zhejiang, China

**Keywords:** MSC-exosomes, skeletal disease, osteoarthritis, osteoporosis, fracture

## Abstract

Skeletal diseases impose a considerable burden on society. The clinical and tissue-engineering therapies applied to alleviate such diseases frequently result in complications and are inadequately effective. Research has shifted from conventional therapies based on mesenchymal stem cells (MSCs) to exosomes derived from MSCs. Exosomes are natural nanocarriers of endogenous DNA, RNA, proteins, and lipids and have a low immune clearance rate and good barrier penetration and allow targeted delivery of therapeutics. MSC-derived exosomes (MSC-exosomes) have the characteristics of both MSCs and exosomes, and so they can have both immunosuppressive and tissue-regenerative effects. Despite advances in our knowledge of MSC-exosomes, their regulatory mechanisms and functionalities are unclear. Here we review the therapeutic potential of MSC-exosomes for skeletal diseases.

## Introduction

The prevalences of musculoskeletal ailments are increasing with the average life expectancy (Pourakbari et al., 2019; Malekpour et al., 2022). Around 1.71 billion people globally have musculoskeletal conditions in 2019, necessitating increasingly complex treatment modalities (Cieza et al., 2021). Osteoarthritis (OA), osteoporosis (OP), intervertebral disc degeneration (IDD), fracture, bone defects, and rheumatoid arthritis (RA) are among the most prevalent skeletal diseases; these not only impose a considerable financial burden on patients but also diminish their overall wellbeing (Li et al., 2018; He et al., 2021; Ding et al., 2023; Torrecillas-Baena et al., 2023). A systematic review pointed out that the global prevalence of OP was reported to be over 23.1% in women (Salari et al., 2021). In China, about 20.6% of females above 40 years old were suffering from OP (Wang et al., 2021). For OA, the mean cost of working-age OA patients is reported to be $14,521 per year. Moreover, the estimated prevalence in adult population is up to 26% by 2040 (Lo et al., 2021). Research in tissue bioengineering has led to the development of liposomes, dendrimers, micelles, and inorganic nanoparticles, which augment the effectiveness of drugs while mitigating their systemic toxicity. However, the clinical application of these agents is hampered by their cytotoxicity and poor biodegradability. Therefore, it is important to develop novel therapeutics that can overcome the above limitations and ameliorate skeletal diseases.

Mesenchymal stem cells (MSCs) have therapeutic potential for skeletal diseases (Wang et al., 2022; Yu et al., 2022; Torrecillas-Baena et al., 2023). The multipotent characteristics of MSCs enable them to undergo self-renewal and differentiate into multiple lineages. Furthermore, they migrate toward injured areas and secrete growth factors, thereby facilitating wound healing (Gholami Farashah et al., 2022; Malekpour et al., 2022). MSC transplantation therapy (MSCT) shows considerable promise for the treatment of diverse ailments. However, the hypopermeability and low blood circulation of bone impedes the application of MSCT for skeletal diseases (Shang et al., 2021). In addition, the survival of engrafted MSCs is poor, and the effects of MSCT are characterized by the paracrine release of cytokines, and exosomes rather than the direct actions of the cells themselves (Vitha et al., 2019; Wang et al., 2022).

**FIGURE 1:**
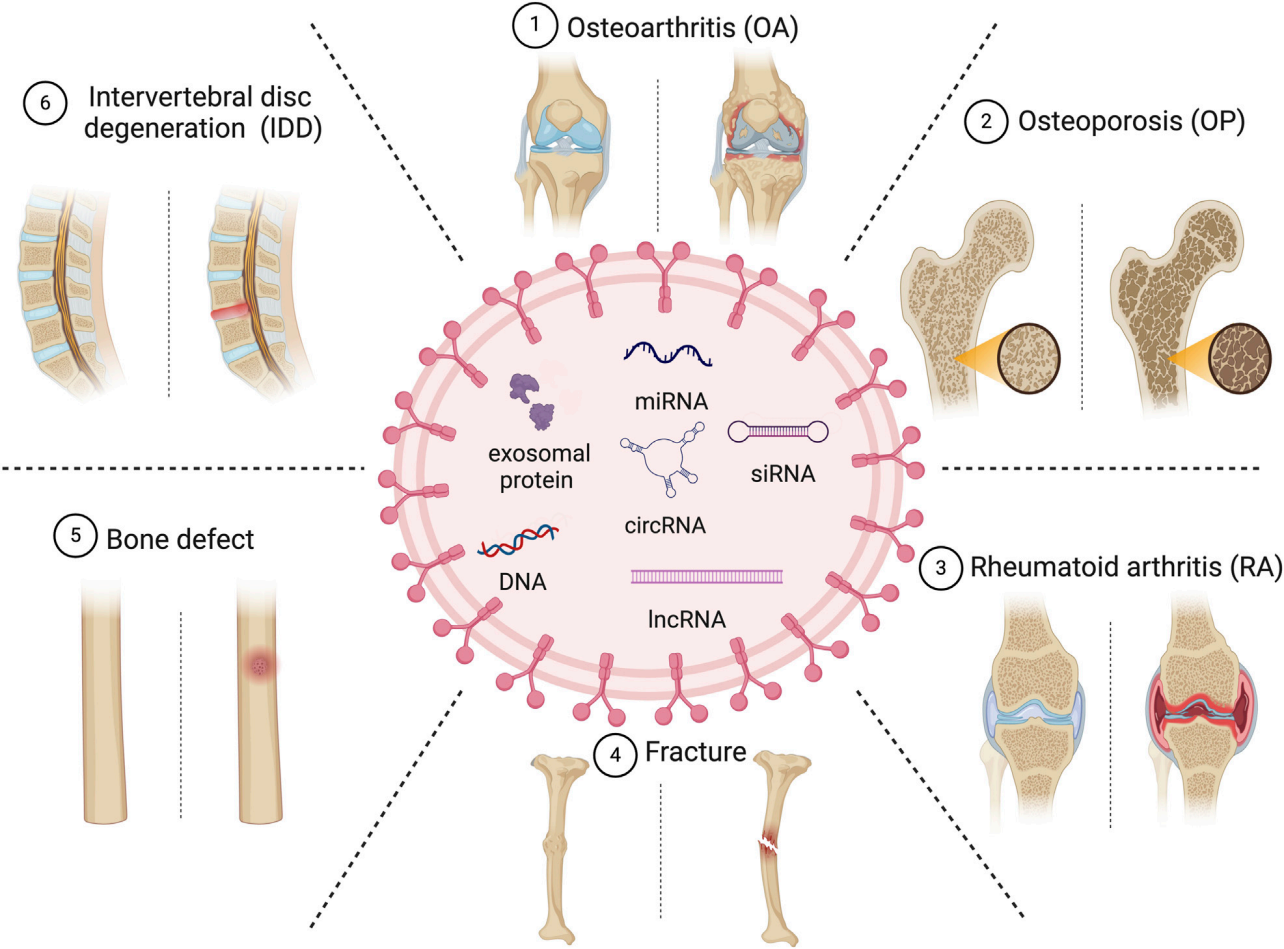
Overview of the therapeutic potential of MSC-exosomes in skeletal diseases.

Exosomes, characterized as scale extracellular vesicles with diameters ranging from approximately 30–150 nm (Kubiatowicz et al., 2022), are released by various cellular entities. They transport biologically active endogenous and exogenous factors such as nucleic acids, proteins, lipids, oligonucleotides, therapeutic RNAs, and small molecules, thereby modulating physiological and pathological processes (Shang et al., 2021; Gholami Farashah et al., 2022). As natural nanocarriers, they transport endogenous factors and have a low immune-clearance rate, good barrier penetration, and allow targeted delivery of therapeutics. MSC-derived exosomes (MSC-exosomes) modulate the functionality of recipient cells by conveying information in the form of constituents of MSCs (Tan et al., 2020; Torrecillas-Baena et al., 2023). Bone marrow-derived MSCs are typically used to produce MSC-exosomes. Compared to MSCT, therapies based on MSC-exosomes have enhanced safety and more convenient storage, transportation, and administration. Consequently, the clinical potential of MSC-exosomes warrants further investigation. The isolation, engineering, cargo loading, and boosting of exosomes have been investigated (Zhang et al., 2019; Wang et al., 2023). This review primarily concentrates on the recent advancements in the utilization of MSC-exosomes for the therapeutic intervention of skeletal disorders including OP, OA, RA, fracture, and IDD. In addition, we discuss barriers to their clinical application (Figure 1).

### The biogenesis and composition of exosomes

**FIGURE 2:**
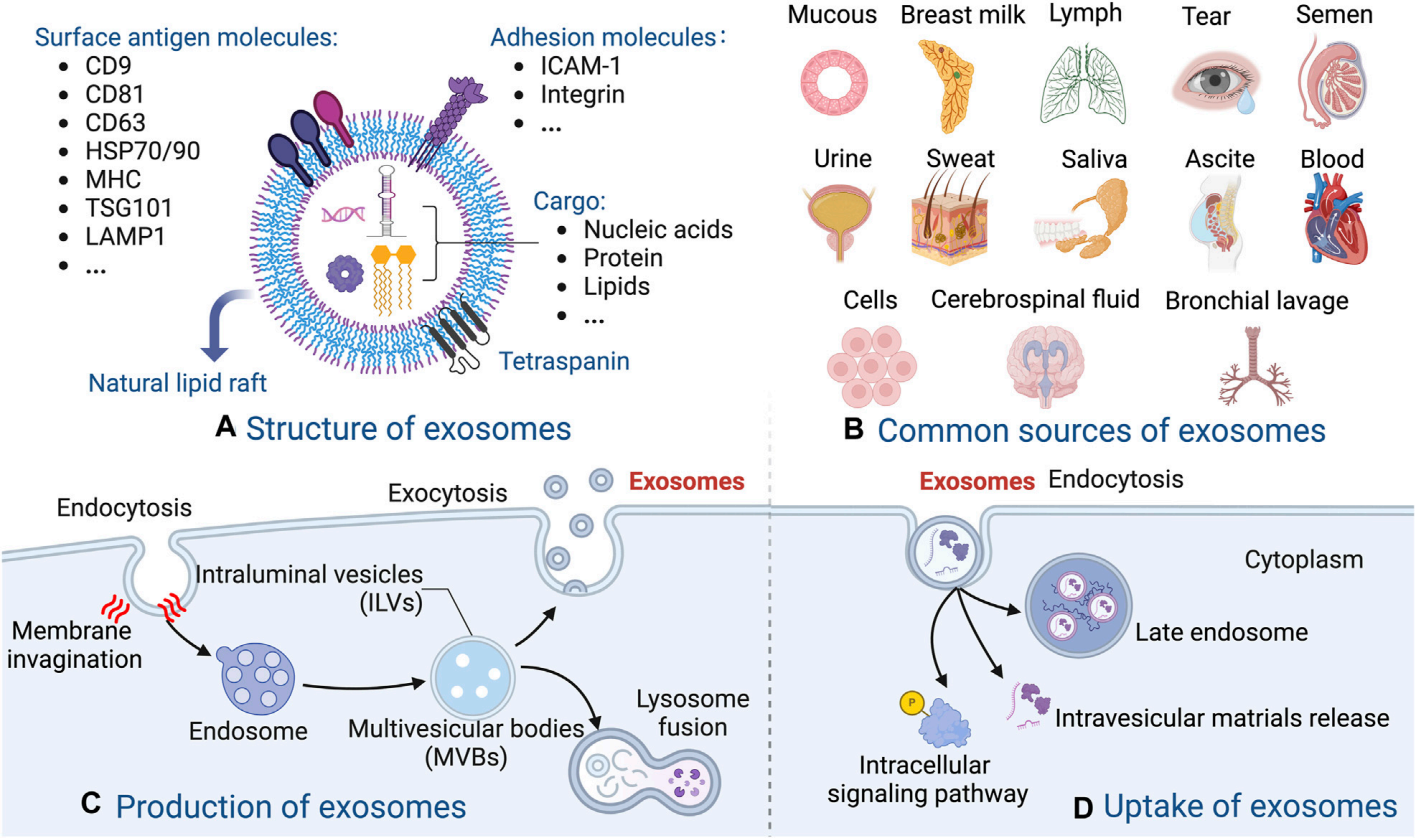
Overview of the biogenesis and composition of exosomes. **(A)** The molecular structure of exosomes. **(B)** The common sources of exosomes. **(C)** The production of exosomes. **(D)** Example of the uptake of exosomes.

Exosome generation involves plasma membrane double invagination and the subsequent synthesis of intracellular multivesicular bodies (MVBs) that harbor intraluminal vesicles (ILVs) (Wang and Thomsen, 2021; Wang et al., 2022). The initial plasma membrane invagination gives rise to a structure that contains cell-surface and extracellular proteins, leading to the *de novo* creation of early sorting endosomes (ESEs), which subsequently become late-sorting endosomes (LSEs) and ultimately generate MVBs. The endoplasmic reticulum also plays a role in the synthesis and content of ESEs. MVBs can undergo fusion with lysosomes, leading to their degradation, resulting in the release of enclosed ILVs as exosomes (Vig and Fernandes, 2022). There are different up-take mechanisms, including fusion, internalization by endocytosis, phagocytosis, etc. (Figures 1, 2).

Exosomes are present in diverse bodily fluids, including blood, saliva, amniotic fluid, hydrocephalus, and urine *et al.*, and serve as vehicles for intercellular information exchange (Behera and Tyagi, 2018; Huang et al., 2022). The markers of exosomes include CD81, CD9, CD63, tumor susceptibility gene 101 (TSG101), heat shock protein 70/90 (HSP70/90), major histocompatibility complex (MHC), and lysosomal-associated membrane protein 1 (LAMP1) et al., which varies among different MSC types. In addition, the cargo of exosomes also shows significant differences, in terms of quantity and diversity, according to the originate cells from which they are derived (Malekpour et al., 2022; Kushioka et al., 2023). Upon binding to recipient cells, exosomes unload their cargo into these cells, thereby mediating intercellular signaling and material exchange, and ultimately modulating the functionality of the recipient cells (Zeng and Xie, 2022). Exosomes, including MSC-exosomes, markedly influence the immune response and inflammation (Huang et al., 2022). The membranes of MSC-exosomes protect their contents, and naturally occurring or artificially altered biomacromolecules on the exosomal surface facilitate the identification of target cells or tissues. Overall, the diagnostic and therapeutic potential are achieved profoundly via substantial transportation of bioactive agents within MSC-exosomes (Bellavia et al., 2018; Li et al., 2018) (Figure 2).

### Therapeutic application of MSC-exosomes for skeletal diseases

#### MSC-exosomes in OA

**FIGURE 3:**
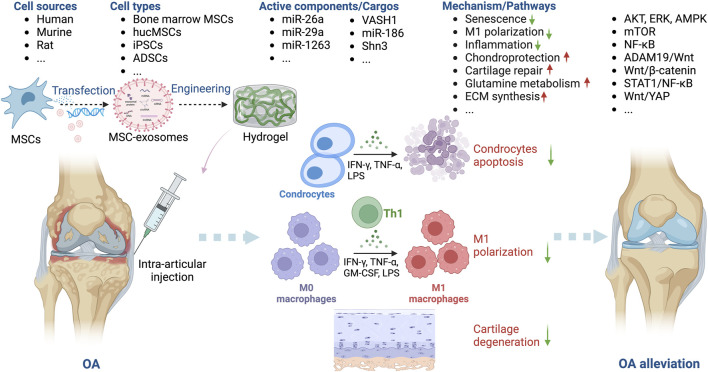
Overview of the therapeutic application of MSC-exosomes in OA, including the cell sources of MSC-exosomes, the active components/cargos, and the possible mechanism/pathways during the alleviation of OA using MSC-exosomes. Up-arrow (red), upregulation; down-arrow (green), downregulation.

OA, the most prevalent chronic disease of the joints, affects a substantial proportion of individuals ≥50 years of age. Given the aging population and the escalating rate of obesity, it is anticipated that its incidence will double over the next three decades (Bao and He, 2021; Cheng et al., 2022). OA is distinguished by the degeneration of cartilage, thickening of subchondral bone, and the development of osteophytes (Chen et al., 2023; Zou et al., 2023). Most interventions aim to manage pain, stiffness, and swelling, and arthroplasty is the only option for late-stage OA. Regrettably, despite the temporary relief provided by physical or drug therapy, restoration of joint function is challenging (Duan et al., 2020; Foo et al., 2021; Ghafouri-Fard et al., 2021). Most research on the clinical potential of MSC-exosomes has been on OA (Yuan et al., 2022; Zeng and Xie, 2022; Zhang et al., 2022; Chen et al., 2023) (Table 1; Figure 3).

**TABLE 1:** Representative *in vivo* studies of MSC-exosomes in OA.

Studies	Sources	Cargos	*In vitro* cells	Conditioning/ Engineering	Animal	OA model	Mechanism
Zhang et al. (2019)	MSCs	NA	Chondrocytes	NA	SD rats	MIA	AKT, ERK, AMPK
Wu et al. (2019)	IPFP-MSCs	NA	Chondrocytes	NA	C57BL/6 mice	DMM	miR-100-5p/mTOR
Liao et al. (2021)	bone marrow MSCs	NA	Chondrocytes	NA	SD rats	ACLT + MMx	NF-κB
Tao et al. (2021)	bone marrow MSCs	miR-361-5p	Chondrocytes	Transfection	Wistar rats	ACLT	NF-κB
Li et al. (2022)	hucMSCs	NA	Chondrocytes	NA	SD rats	ACLT + MMx	NA
Cao et al. (2023)	hucMSCs	NA	Chondrocytes	Microgels	Rats	ACLT	Senescence alleviation
Kong et al. (2023)	SMSCs	miR-320c	Chondrocytes	Transfection	SD rats	DMM	ADAM19/Wnt
Li et al. (2023)	ADSCs	miR-376c-3p	SFs	Transfection	SD rats	MIA	Wnt/β-catenin
Xu and Xu (2021)	bone marrow MSCs	miR-326	Chondrocytes	Transfection	SD rats	MIA	HDAC3; STAT1/NF-κB
Zhang et al. (2020)	bone marrow MSCs	NA	Chondrocytes/RAW264.7	NA	SD rats	ACLT + MMx	Macrophage polarization
Xu et al. (2021)	SF-MSCs	Kartogenin	DCs	Transfection	SD rats	DMM	NA
Jin et al. (2021)	bone marrow MSCs	NA	Chondrocytes	NA	SD rats	ACLT + DMM	lncRNA MEG-3/Senescence
Tao et al. (2017)	bone marrow MSCs	miR-140-5p	Chondrocytes	Transfection	SD rats	ACLT + DMM	Wnt/YAP
Wang et al. (2017)	ESC-MSCs	NA	Chondrocyte	NA	C57BL/6 J mice	DMM	NA
Jiang et al. (2021)	bone marrow MSCs	NA	Chondrocytes	NA	SD rats	ACLT	NA
Xu et al. (2022)	ADSCs	NA	Chondrocytes	NA	SD rats	ACLT	NA

IPFP-MSCs, infrapatellar fat pad MSCs; SMSCs, synovial MSCs; SF, synovial fluid; ESC-MSCs, embryonic stem cell-induced MSCs; DMM, destabilization of the medial meniscus; SFs, synovial fibroblasts; ACLT, anterior cruciate ligament transection; DCs, dendritic cells; MIA, monosodium iodoacetate; MMx, medial meniscus resection; HDAC3, histone deacetylase 3.

Exosomes derived from bone marrow MSCs and those from adipose stem cells (ADSCs), infrapatellar fat pad (Wu et al., 2019), human umbilical cord (Li et al., 2022; Cao et al., 2023), synovial fluid (Xu et al., 2021), embryonic stem cells (Wang et al., 2017), and synovial membrane (Tao et al., 2017) have been used to generate exosomes for the treatment of OA. Among these sources, ADSCs and bone marrow-derived MSCs are relatively easy to obtain, which may be conducive to future therapeutical applications. Cosenza *et al.* showed that MSC-exosomes and MSC-derived microparticles have similar *in vitro* and *in vivo* chondroprotective effects in OA, thus reproducing the main therapeutic effects of bone marrow MSCs (Cosenza et al., 2017). MSC-exosomes protect cartilage and bone by inhibiting catabolic and inflammatory cytokines, suppressing macrophage activation, and preventing chondrocyte apoptosis (Huang et al., 2022; Kwon et al., 2022). Zhu *et al.* compared induced MSC (iMSC) and synovial MSC (SMSC) exosomes in mice OA model and found that the former had the greatest therapeutic effect in OA. The OARSI score of OA samples was significantly decreased after iMSC-exosomes treatment (Zhu et al., 2017).

Intra-articular injection is typically used to introduce MSC-exosomes into patients with OA (Tao et al., 2017; Huang et al., 2022; Kwon et al., 2022; Li et al., 2022; Cao et al., 2023). MSC-exosomes ameliorate OA by inhibiting inflammation, alleviating senescence, and protecting chondrocytes via the signal transducers and activators of transcription 1 (STAT1), protein kinase B (AKT), extracellular signal-regulated kinase (ERK), AMP-activated protein kinase (AMPK), mammalian target of rapamycin (mTOR), nuclear factor-kappa B (NF-κB), ADAM metallopeptidase domain 19 (ADAM19)/Wnt, and Wnt/yes-associated protein (YAP) signaling pathways (Song et al., 2021; Rosini et al., 2023; Wang et al., 2023). Zhang *et al.* reported that bone marrow-derived MSC-exosomes delay OA progression by modulating macrophage polarization (Zhang et al., 2020). Liao *et al.* showed that low-intensity pulsed ultrasound (LIPUS) strengthens the effect of bone marrow MSC-exosomes on cartilage regeneration in OA by strengthening the inhibition of NF-κB pathway-mediated inflammation and enhancing cartilage matrix synthesis (Liao et al., 2021). In addition, stimulation with a 75 Hz pulsed electromagnetic field promotes the ADSC-exosome–mediated suppression of inflammation and protection of cartilage (Xu et al., 2022).

Several OA studies have combined bioengineering approaches, typically biomaterials, with MSC-exosomes. Zeng *et al.* constructed a mussel-inspired multifunctional hydrogel system for codelivery of MSC-exosomes and icariin. MSC-exosomes enhanced the uptake of icariin by chondrocytes by at least twofold, thereby promoting cartilage regeneration in a papain-induced OA model (Zeng et al., 2023). Cao *et al.* engineered human umbilical cord MSC (hucMSC)-exosomes with a two-phase microgel targeting chondrocytes; the hucMSC-exosomes rejuvenated OA chondrocytes (Cao et al., 2023). Others have reported similar findings (Zhang et al., 2022; Pang et al., 2023), suggesting that combinations of novel biomaterials and MSC-exosomes have potential as cell-free therapeutics for OA.

#### MSC-exosomes in OP

Under normal physiological conditions, the equilibrium between bone resorption and bone formation preserves the integrity and quality of bone tissue. This delicate balance is disturbed in a number of bone disorders. OP, one of the most prevalent skeletal disorders worldwide, disproportionately affects the elderly, particularly women (Tan et al., 2020; Xie et al., 2020). Hormonal, nutritional, behavioral, and genetic factors may contribute to its development and progression, although aging and deficiencies in estrogen are the primary causes. Perturbations in bone metabolism, such as an imbalance in the activities of osteoclasts (OCs) and osteoblasts (OBs), are also implicated in its pathogenesis. During recovery from OP, OBs secrete osteoids to facilitate bone regeneration (Li et al., 2018; Yang et al., 2022). Currently available treatments (including anti-resorptive and anabolic drugs) control OP by promoting bone formation, impeding adipocyte development, or inhibiting OC differentiation. However, the potential adverse effects, which include fever, nausea, bone pain, and cancer, are nonnegligible (Duan and Guan, 2021; Li et al., 2022; Huo et al., 2023).

**FIGURE 4:**
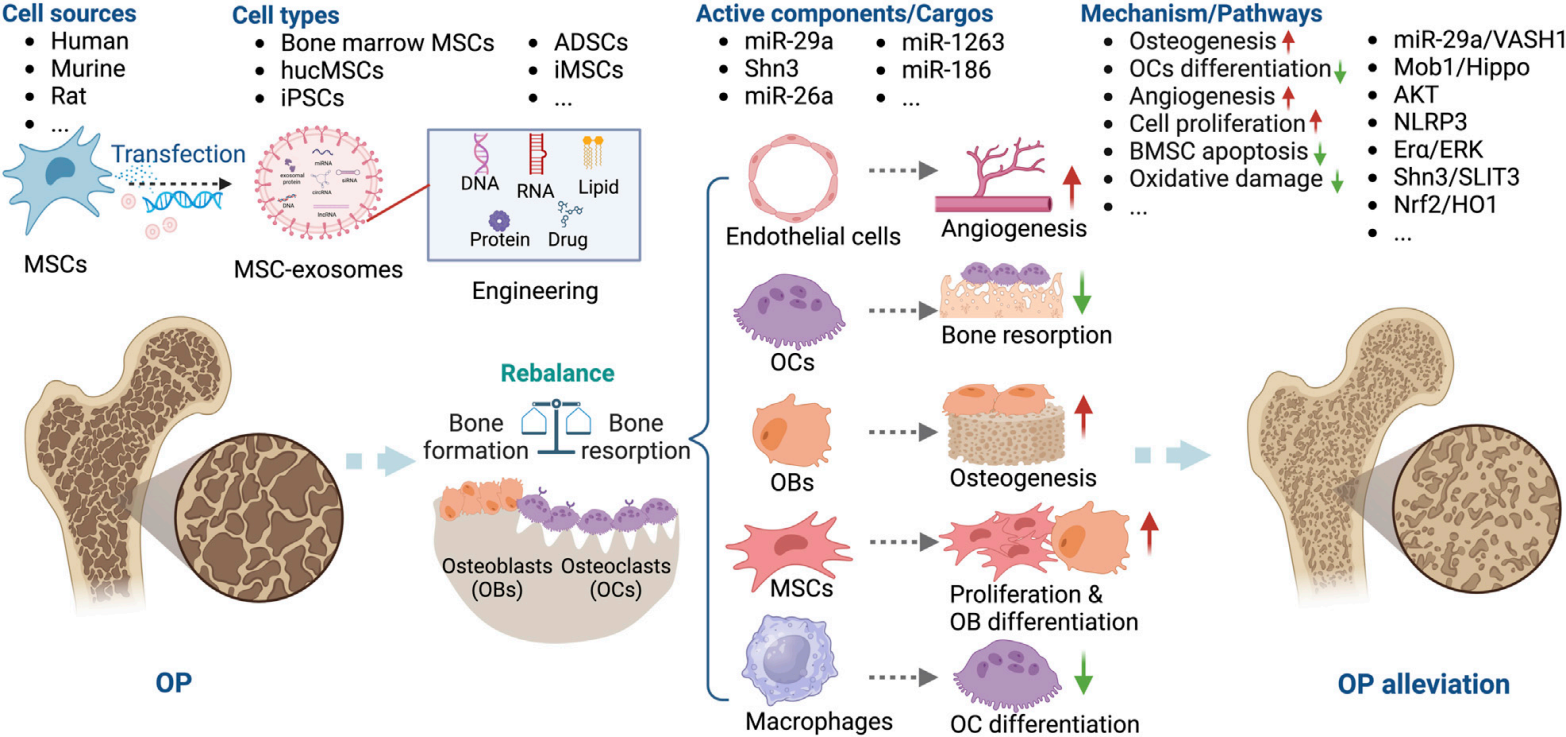
Overview of the therapeutic application of MSC-exosomes in OP, including the cell sources of MSC-exosomes, the active components/cargos, and the possible mechanism/pathways during the alleviation of OP using MSC-exosomes. Up-arrow (red), upregulation; down-arrow (green), downregulation.

MSCs can differentiate into OBs and produce extracellular matrix (ECM), thereby promoting bone formation. Their ability to sustain bone homeostasis declines with aging, menopause, and ovariectomy (OVX), resulting in the accumulation of bone mineral adipocytes, ultimately leading to OP (Behera and Tyagi, 2018; Zeng and Xie, 2022; Ding et al., 2023). OVX-induced OP (Luo et al., 2019; Yahao and Xinjia, 2021; Cui et al., 2022; Qi et al., 2023), senile OP (SOP) (Lu et al., 2020), disuse OP (DOP) (Yang et al., 2020), glucocorticoid-induced OP (GIOP) (Yao et al., 2023), and diabetic OP (Zhang et al., 2021) are the models typically used to assess the therapeutic effect of MSC-exosomes in OP (Table 2). Bone marrow-derived MSCs are usually used to generate exosomes (Luo et al., 2019; Lu et al., 2020; Li et al., 2021; Qi et al., 2023), as are hucMSCs (Yang et al., 2020), ADSCs (Yao et al., 2023), and induced pluripotent stem cells (iPSCs) (Cui et al., 2022). The effects of MSC-exosomes in OP is mediated by enhancement of osteogenesis and angiogenesis, possibly via the vasohibin 1 (VASH1) (Lu et al., 2020), Mob1/Hippo (Yang et al., 2020), NOD-like receptor thermal protein domain associated protein 3 (NLRP3) (Zhang et al., 2021), schnurri-3 (Shn3)/Slit guidance ligand 3 (SLIT3) (Cui et al., 2022), and nuclear factor erythroid 2-related factor 2 (Nrf2)/heme oxygenase-1 (HO1) (Yao et al., 2023) signaling pathways (Figure 4). Zuo *et al.* reported that bone marrow-derived MSC-exosomes increased the β-catenin expression of recipient bone marrow-derived MSCs and restored the adipogenesis–osteogenesis balance, thereby alleviating radiation-induced bone loss (Zuo et al., 2019). ADSC exosomes alleviate streptozotocin (STZ)-induced diabetic OP by suppressing the NLRP3 inflammasome activation (Zhang et al., 2021).

**TABLE 2:** Representative *in vivo* studies of MSC-exosomes in OP.

Studies	Sources	Cargos	*In vitro* cells	Conditioning/ Engineering	Animal	Model	Injection	Mechanism
Luo et al. (2019)	bone marrow MSCs	antagomiR-26a	bone marrow MSCs, RAW264.7	Aptamer	C57BL/6 mice	OVX	*i.v*	NA
Lu et al. (2020)	bone marrow MSCs	miR-29a	bone marrow MSCs; HUVECs	Transfection	C57BL/6 mice	SOP	*i.v*	miR-29a/VASH1
Yang et al. (2020)	hucMSCs	miR-1263	bone marrow MSCs	Transfection	SD rats	DOP	*i.m*	miR-1263/Mob1/Hippo
Zhang et al. (2021)	ADSCs	NA	BMMs	NA	SD rats	Diabetic OP	*i.v*	NLRP3
Qi et al. (2023)	bone marrow MSCs	NA	MG-63	NA	SD rats	OVX	*i.v*	Erα/ERK
Li et al. (2021)	bone marrow MSCs	NA	bone marrow MSCs	NA	SD rats	OVX	*i.v*	miR-186/Hippo
Cui et al. (2022)	iPSCs	siShn3	MC3T3-E1/bone marrow MSCs	Bone-targeting	C57BL/6 mice	OVX	*i.v*	Shn3/SLIT3
Yahao and Xinjia (2021)	hucMSCs	NA	OBs	Osteogenic differentiation	C57BL/6 mice	OVX	*i.p*	NA
Yao et al. (2023)	ADSCs	NA	MC3T3-E1	NA	SD rats	GIOP	*i.v*	Nrf2/HO1

BMMs, bone marrow-derived macrophages; HUVECs, human umbilical vein endothelial cells; SD rats, Sprague Dawley rats; Erα, estrogen receptor α; *i. v*., intravenous injection; *i. p*., intraperitoneally injection; *i. m*., intramuscular injection; NA, not applicable.

Several miRNAs, including miR-26a, miR-29a, miR-1263, and miR-186, are implicated in the pathogenesis of OP (Lai et al., 2022). MSC-exosomes have been used to deliver miRNAs or anti-miRNAs for the treatment of OP. Bioengineering methods such as the use of aptamers and OB differentiation preconditioning can be used to amplify the effects of MSC-exosomes in OP. Luo *et al.* (Luo et al., 2019) showed that bone marrow MSC-exosomes administered intravenously did not ameliorate OVX-induced OP in a mouse model. They conjugated a bone marrow MSC-specific aptamer to the surface of bone marrow-derived MSC-exosomes to target bone marrow, which enhanced bone regeneration in OVX mice. Similarly, Cui *et al.* functionalized iPSC exosomes by modifying a bone-targeting peptide. The exosomes were loaded with the Shn3 siRNA and showed therapeutic potential for OP by enhancing bone and vessel formation and inhibiting OCs (Cui et al., 2022). Ge *et al.* compared the functions of hucMSC-exosomes from normal culture and those produced via osteogenic differentiation preconditioning. Interestingly, exosomes produced in an hucMSC/OB coculture system showed greater promotion of osteogenesis, confirming the importance of preconditioning for the effectiveness of MSC-exosomes in OP (Yahao and Xinjia, 2021). Consequently, MSC-exosomes have potential as a novel therapeutic strategy for OP.

#### MSC-exosomes in RA

RA is a chronic autoimmune disease that affects up to 2.5% of the population in each country, causing cartilage destruction and bone erosion (You et al., 2021). Current treatments focus on suppressing inflammation, but side effects like bone loss and long-term toxicities remain a challenge (Xu et al., 2022). Defective immune regulation leads to autoreactive T and B lymphocytes activation and differentiation, leading to the produce of autoreactive antibodies, activation of inflammatory responses and cartilage degeneration (Cosenza et al., 2017; Chang et al., 2022; Heydari et al., 2023). MSC-exosomes have not only anti-inflammatory but also immunomodulatory effects, which is why they help mitigate joint destruction (Heydari et al., 2023; Zhao et al., 2023).

Collagen-induced arthritis (CIA) (Chen et al., 2018; You et al., 2021) and Freund’s adjuvant-induced arthritis (FAIA) (Chang and Kan, 2021) in DBA/1J mice, C57BL/6 mice, and rats are frequently used animal models of RA. Dermal microvascular endothelial cells (DMECs) (Zhang et al., 2021), fibroblast-like synoviocytes (FLSs) (Ma et al., 2022), and lymphocytes (Tian et al., 2022) have been used to evaluate the role of MSC-exosomes in RA. MSC-exosomes have been shown to modulate the pathogenesis of RA (You et al., 2021; Ma et al., 2022; Rui et al., 2023). You *et al.* modified the surface of ADSC exosomes to reprogram macrophages. After intravenous injection (*i.v.*), the engineered ADSC exosomes accumulated in diseased joints and modulated the synovial microenvironment, thereby having a marked anti-inflammatory effect in RA (You et al., 2021). Rui *et al.* synthesized silk fibroin hydrogel encapsulated with olfactory ecto-MSCs (OEMSCs)-derived exosomes; their implantation altered T follicular helper cell polarization by regulating programmed cell death ligand 1 (PD-L1), thereby alleviating synovial inflammation and joint destruction (Rui et al., 2023) (Table 3).

**TABLE 3:** Representative *in vivo* studies of MSC-exosomes in RA.

Studies	Sources	Cargos	*In vitro* cells	Conditioning/ Engineering	Animal	Injection	Mechanism
Chen et al. (2018)	bone marrow MSCs	miR-150-5p	FLSs/HUVECs	Transfection	DBA/1 mice	*i.p*	MMP14/VEGF
Cosenza et al. (2018)	bone marrow MSCs	NA	T and B cells	NA	DBA/1mice	*i.v*	Immunosuppression
Tian et al. (2022)	GMSCs	NA	T cells/GMSCs	NA	DBA/1J mice	*i.v*	IL-17RA/Act1/TRAF6/NF-κB
Zhang et al. (2021)	SMSCs	circEDIL3	SMSCs/FLSs/ DMECs	Transfection	DBA/1J mice	*i.a*	circEDIL3/miR-485-3p/PIAS3/STAT3/VEGF
Rui et al. (2023)	OEMSCs	NA	bone marrow MSCs/T cells	Hydrogel	DBA/1J mice	*i.a*	PD-L1/PI3K/AKT
Ma et al. (2022)	bone marrow MSCs	miR-205-5p	FLSs	Chondrogenesis/ Transfection	C57BL/6 mice	*i.d*	MAPK; NF-κB
Huang et al. (2022)	hucMSCs	miR-140-3p	RASFs	Transfection	Wistar rats	NA	miR-140-3p/SGK1
Xu et al. (2022)	bone marrow MSCs	FGL1	FLSs	Transfection	SD rats	NA	NF-κB
Tavasolian et al. (2020)	ADSCs	miR-146a/ miR-155	Splenocytes	Transfection	C57BL/6 mice	NA	Autoimmune response
Fu et al. (2022)	hucMSCs	NA	NA	NA	DBA/1J mice	*i.v*	Th1/Th17/Treg balance

*i.a*., intra-articular injection; *i. d*., intradermal injection; GMSCs, gingival MSCs; RASFs, RA, synovial fibroblasts; SGK1, serum and glucocorticoid-regulated kinase 1.

**FIGURE 5:**
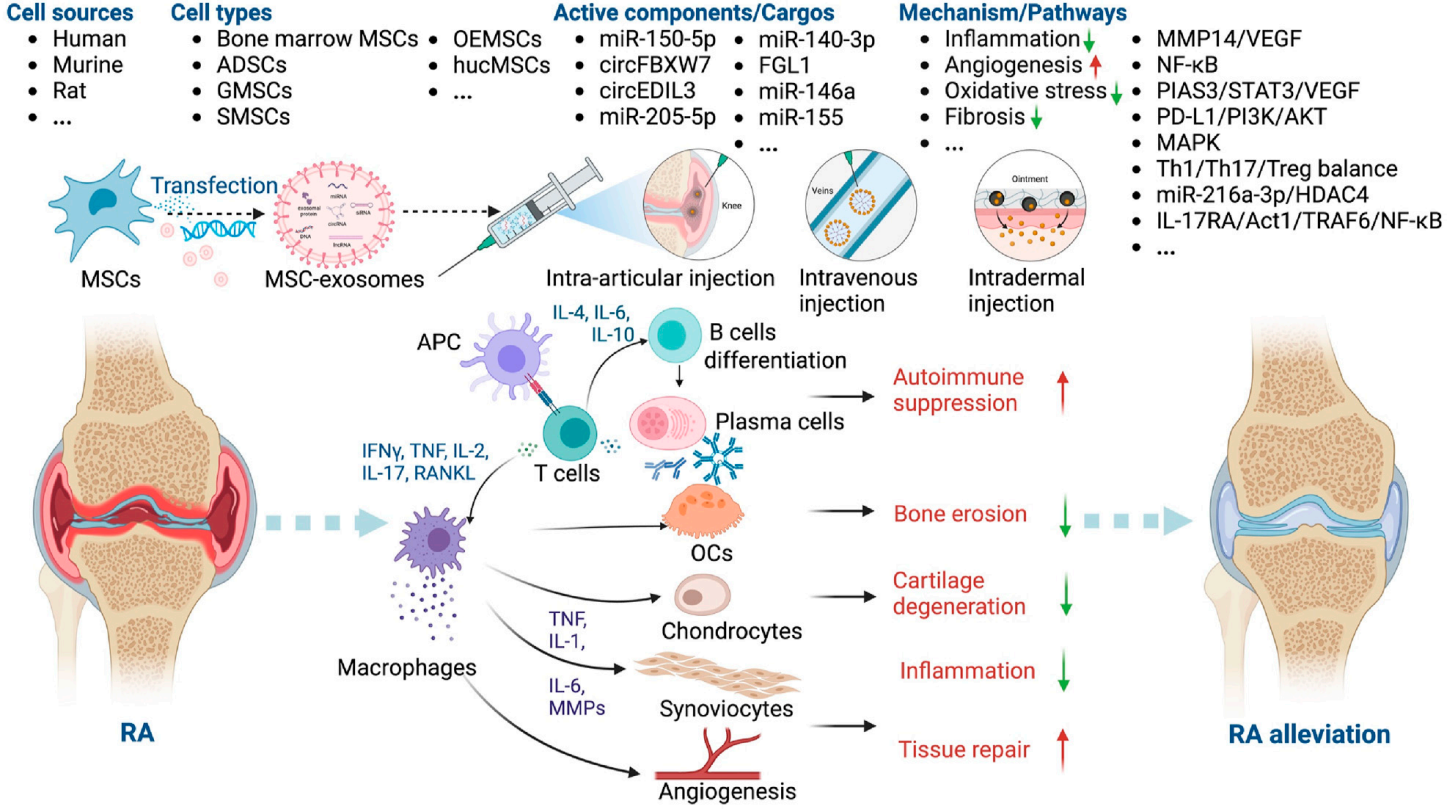
Overview of the therapeutic application of MSC-exosomes in RA, including the cell sources of MSC-exosomes, the active components/cargos, and the possible mechanism/pathways during the alleviation of RA using MSC-exosomes. Up-arrow (red), upregulation; down-arrow (green), downregulation.

The potential mechanisms underlying the therapeutic effects of MSC-exosomes in RA include modification of macrophage heterogeneity and autoimmunity. These effects are mediated by regulation of the matrix metalloproteinase 14 (MMP14)/vascular endothelial growth factor (VEGF) (Chen et al., 2018), interleukin 17 receptor A (IL-17RA)/NF-κB activator 1 (Act1)/TNF receptor associated factor 6 (TRAF6)/NF-κB (Tian et al., 2022), protein inhibitor of activated STAT3 (PIAS3)/STAT3/VEGF (Zhang et al., 2021), and PD-L1/phosphoinositide 3-kinase (PI3K)/AKT (Rui et al., 2023) signaling pathways. In addition, miR-150-5p (Chen et al., 2018), circFBXW7 (Chang and Kan, 2021), fibrinogen-like protein 1 (FGL1) (Xu et al., 2022), miR-146a, and miR-155 (Tavasolian et al., 2020) modulate the therapeutic effect of MSC-exosomes in RA. Huang *et al.* showed that miR-140-3p alleviates the inflammatory response of RA synovial fibroblasts (Huang et al., 2022). Given their immunoregulatory, chondroprotective, and regenerative activities, these factors likely contribute to the protective effects of MSC-exosomes in RA (Figure 5).

#### MSC-exosomes in fracture and bone-defect healing

Fractures and bone defects are common musculoskeletal issues, and approximately 5%–10% of patients experience delayed union or nonunion as a result of inadequate bone regeneration (He et al., 2021; Smolinska et al., 2023). Bone regeneration involves various cell types, including OBs, OCs, endothelial cells, chondrocytes, and MSCs. Bone regeneration can be mediated by intramembranous ossification or endochondral ossification. MSC-derived OBs directly contribute to the calcification of bone via intramembranous osteogenesis, whereas endochondral ossification is an intricate process regulated by different cells, including chondrocytes. MSCs promote bone regeneration, an effect mediated by several key factors, including exosomes (Liu et al., 2020; Huang et al., 2022; Smolinska et al., 2023). Indeed, MSC-exosomes promote the repair of fractures (Table 4) and bone defects (Table 5).

**TABLE 4:** Representative *in vivo* studies of MSC-exosomes in fracture.

Studies	Sources	Cargos	*In vitro* model	Conditioning/ Engineering	Animal	Injection	Mechanism
Furuta et al. (2016)	bone marrow MSCs	NA	NA	NA	C57BL/6 mice/ CD9^−/−^ mice	Local	NA
Zhang et al. (2020)	bone marrow MSCs	NA	HUVECs/MC3TE-E1	NA	Wistar rats	Local	BMP-2/Smad1/Runx2; HIF-1α/VEGF
Liu et al. (2020)	hucMSCs	miR-126 inhibitor	HUVECs/FOB 1.19	Hypoxic/Transfection	Mice	Local	SPRED1/Ras/Erk
Luo et al. (2019)	bone marrow MSCs	miR-26a inhibitor	bone marrow MSCs	Aptamer	C57BL/6 mice	*i.v*	NA
Zhang et al. (2019)	hucMSCs	NA	OBs/HUVECs	Hydrogel	Wistar rats	Local	HIF-1α/VEGF
Yu et al. (2021)	bone marrow MSCs	miR-136-5p	MC3T3-E1	Transfection	C57BL/6 mice	*i.v*	LRP4/Wnt/βcatenin
Huang et al. (2021)	bone marrow MSCs	NA	bone marrow MSCs	NA	C57BL/6 mice	Local	miR-19b/WWP1/Smurf2/ KLF5/β-catenin
Xu et al. (2020)	bone marrow MSCs	NA	bone marrow MSCs	Transfection	SD rats	Local	miR-128-3P/Smad5
Li et al. (2023)	bone marrow MSCs	LncTUG1	bone marrow MSCs	Transfection	C57BL/6 mice	Local	miR-22-5p/Anxa8
Zhou et al. (2019)	hucMSCs	NA	NA	Hydrogel	SD rats	Local	Wnt/β-catenin
Zhang et al. (2023)	ADSCs	NA	bone marrow MSCs	NA	SD rats	Local	Wnt3a/β-Catenin

SPRED1, Sprouty related EVH1 domain containing 1; KLF5, KLF, transcription factor 5; *i. v*., intravenous injection.

**TABLE 5:** Representative *in vivo* studies of MSC-exosomes in bone defect.

Studies	Sources	Cargos	*In vitro* model	Conditioning/ Engineering	Animal	Bone defect model	Mechanism
Chen et al. (2019)	ADSCs	miR-375	bone marrow MSCs	Transfection/hydrogel	SD rats	Calvarial	NA
Xu et al. (2023)	bone marrow MSCs	NA	bone marrow MSCs	Hydrogel	SD rats	Spinal column	NA
Qi et al. (2016)	iMSCs	NA	bone marrow MSCs	Scaffolds	SD rats	Calvarial	NA
Sun et al. (2023)	hucMSCs	NA	HUVECs	Scaffold	SD rats	Alveolar bone	NA
Ma et al. (2022)	bone marrow MSCs	NA	bone marrow MSCs	Peptides/hydrogel	SD rats	Calvarial	NA
Takeuchi et al. (2019)	bone marrow MSCs	NA	bone marrow MSCs	Scaffold	Wistar rats	Calvarial	NA
Takeuchi et al. (2019)	hucMSCs	NA	bone marrow MSCs	Scaffold	Wistar rats	Calvarial	miR-21/NOTCH1/DLL4
Swanson et al. (2020)	DPSCs	NA	bone marrow MSCs	Scaffold	C57BL/6 mice	Calvarial	NA
Zhang et al. (2016)	iMSCs	NA	bone marrow MSCs	β-TCP scaffold	SD rats	Calvarial	PI3K/AKT
Wang et al. (2022)	bone marrow MSCs	NA	bone marrow MSCs	OB induction/scaffold	C57BL/6 mice	Calvarial	NA
Ying et al. (2020)	bone marrow MSCs	HIF-1α	bone marrow MSCs	Scaffold	SD rats	Calvarial	NA
Kang et al. (2022)	ADSCs	NA	bone marrow MSCs; HUVECs	Scaffold	SD rats	Calvarial	NA
Liang et al. (2019)	bone marrow MSCs	NA	HUVECs	DMOG stimulated/scaffold	SD rats	Calvarial	AKT/mTOR
Li et al. (2018)	ADSCs	NA	bone marrow MSCs	Scaffold	BALB/C mice	Calvarial	NA
Wang et al. (2020)	hucMSCs	NA	OPCs; HUVECs	Scaffold	SD rats	Femoral condyle	NA

DPSCs, dental pulp stem cells; DMOG, dimethyloxaloylglycine; OPCs, mouse osteoblast progenitor cells; NOTCH1, neurogenic locus notch homolog protein 1; DLL4, delta-like 4.

**FIGURE 6:**
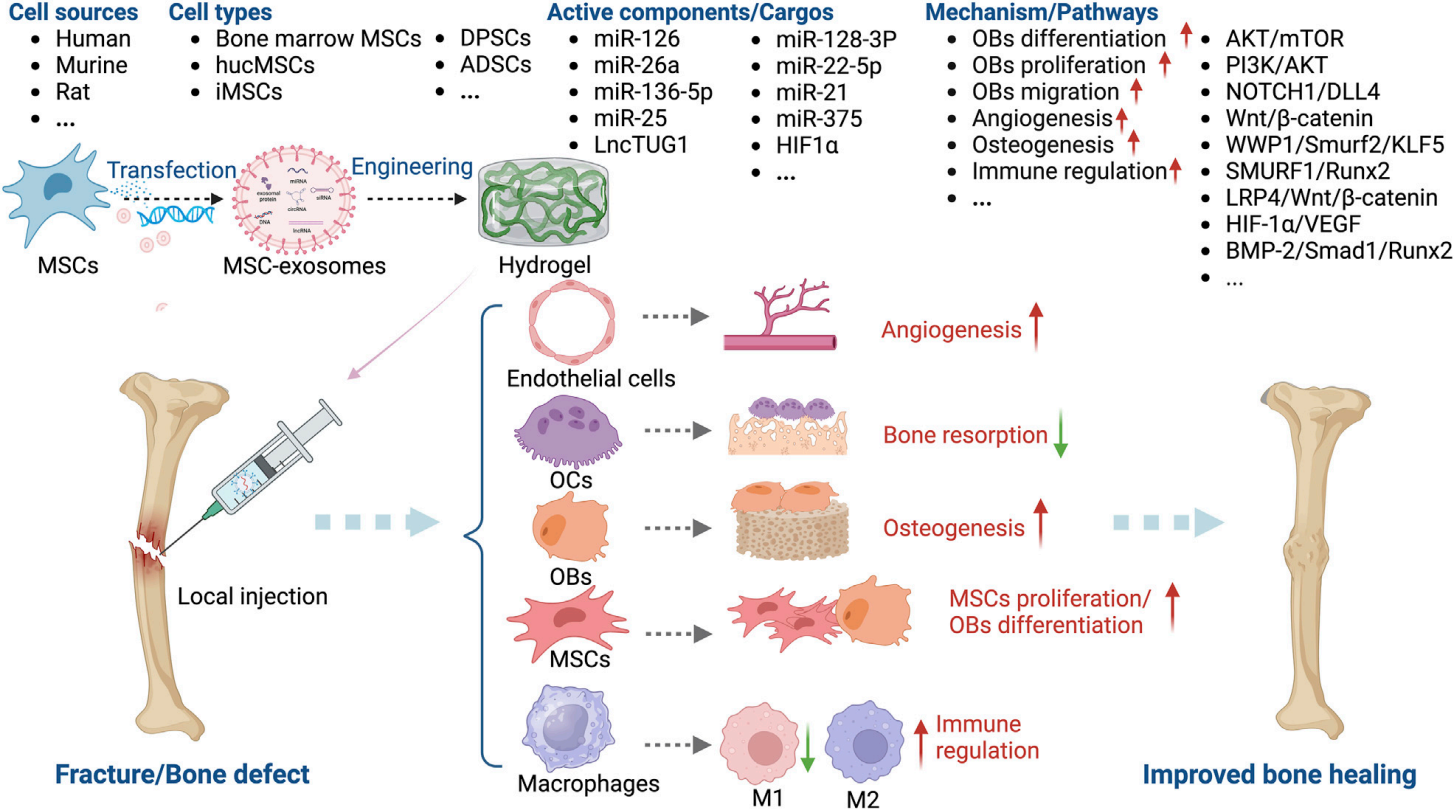
Overview of the therapeutic application of MSC-exosomes in fracture/bone defect, including the cell sources of MSC-exosomes, the active components/cargos, and the possible mechanism/pathways during the alleviation of fracture/bone defect using MSC-exosomes. Up-arrow (red), upregulation; down-arrow (green), downregulation.

Angiogenesis and ossification are prerequisites for bone healing, and MSC-exosomes augment OB differentiation and mineral deposition, thus facilitating angiogenesis, via the bone morphogenetic protein 2 (BMP-2)/Smad1/Runx2, hypoxia-inducible factor α (HIF-1α)/VEGF (Zhang et al., 2020), LDL receptor related protein 4 (LRP4)/Wnt/β-catenin (Yu et al., 2021), W domain-containing E3 ubiquitin protein ligase 1 (WWP1)/Smad ubiquitin regulatory factor 2 (Smurf2)/KLF transcription factor 5 (KLF5)/β-catenin (Huang et al., 2021) signaling pathways. Moreover, the cytokines, miRNAs, and lncRNAs such as miR-136-5p (Yu et al., 2021), lncTUG1 (Li et al., 2023), and monocyte chemoattractant protein-1 (MCP-1) (Furuta et al., 2016) delivered by MSC-exosomes promote the healing of fractures and bone defects (Figure 6).

Furuta *et al.* investigated MSC-exosomes in a CD9^−/−^ mouse femur fracture model and found that bone union was significantly accelerated by MSC paracrine signaling (Furuta et al., 2016). Liu *et al.* showed that exosomes from hypoxia-preconditioned hucMSCs have a greater effect on fracture healing than those from normoxia-preconditioned hucMSCs, emphasizing the importance of mimicking normal physiological conditions (Liu et al., 2020). Unlike OP, OA, and RA, MSC-exosomes for the healing of fractures and bone defects are typically administered in a hydrogel-based local implant or by local injection. Ma *et al.* combined small intestinal submucosa hydrogels with bone marrow-derived MSC-exosomes and fusion peptides to enhance the osteogenesis-promoting role of exosomes in a calvarial defect model (Ma et al., 2022). Chen *et al.* incorporated miR-375-carrying ADSC exosomes in a hydrogel to achieve the slow and controlled release of miR-375, which had a marked bone-healing effect in a rat model of a calvarial defect (Chen et al., 2019).

#### MSC-exosomes in IDD

The intervertebral disc (IVD), including the nucleus pulposus (NP) and annulus fibrosus (AF), is an important load-bearing component of the spinal column. The accumulation of advanced glycation end products (AGEs) causes endoplasmic reticulum (ER) stress in the IVD. Subsequently, IDD is initiated by the apoptosis of NP cells (NPCs) and increased pro-inflammatory cytokine production and disruption of the ECM (Widjaja et al., 2022; Xia et al., 2022). IDD is the main reason for low back pain in over 90% of people over 50 (Krut et al., 2021). Surgical therapy and pain relief medication are the main treatments, but their effectiveness is uncertain (Xiao et al., 2022).

MSCs can enhance the viability of disc cells and thus impede IDD progression, typically by modulating the levels of MMP12 and HSP47 (Leung et al., 2014). MSC-exosomes modulate the inflammatory response of NPCs, suppressing their apoptosis and upregulating ECM synthesis (Bhujel et al., 2022; Distefano et al., 2022; Hu et al., 2023) (Table 6).

**TABLE 6:** Representative *in vivo* studies of MSC-exosomes in IDD.

Studies	Sources	Cargos	*In vitro* model	Pre-conditioning Engineering	Animal	Model	Injection	Mechanism
Liao et al. (2019)	bone marrow MSCs	NA	NPCs	NA	SD rats	AGEs	*i.d.s*	AKT and ERK
Zhang et al. (2020)	MSCs	miR-410	NPCs	Transfection	C57BL/6 mice	Puncture	*i.v*	miR-410/NLRP3
Xie et al. (2020)	MSCs	antagomir-31-5p	EPCs	Transfection	SD rats	Puncture	*i. s. e*	miR-31-5p/ATF6/ER stress
Xia et al. (2019)	bone marrow MSCs	NA	NPCs	NA	Rabbit	Puncture	*i.d.s*	ROS/TXNIP/NLRP3
Yu et al. (2023)	ESCs	miR-302c antagomir	NPCs	Transfection	SD rats	Puncture	*i.d.s*	miR-302c/NLRP3
Li et al. (2022)	bone marrow MSCs	siCAHM	THP-1 cells/NPCs	Transfection	SD rats	Puncture	*i.d.s*	macrophage polarization
Guan et al. (2023)	bone marrow MSCs	NA	NPCs	Hydrogel	SD rats	Puncture	*i.d.s*	senescence alleviation
Xiao et al. (2022)	bone marrow MSCs	NA	NPCs	NA	SD rats	Puncture	*i.d.s*	AKT/mTOR/autophagy

ESCs, embryonic stem cells; EPCs, endplate chondrocytes; ATF6, activating transcription factor 6; *i.d.s*., intradiscal injection; *i. s. e*., sub-endplate injection.

**FIGURE 7:**
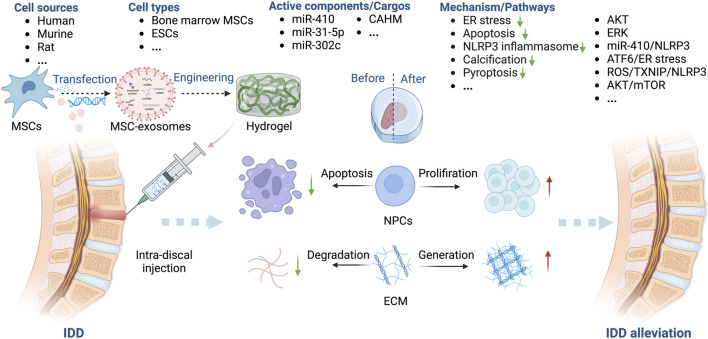
Overview of the therapeutic application of MSC-exosomes in IDD, including the cell sources of MSC-exosomes, the active components/cargos, and the possible mechanism/pathways during the alleviation of IDD using MSC-exosomes. ECM: extracellular matrix. Up-arrow (red), upregulation; down-arrow (green), downregulation.

The key factors for MSC-exosome treatment of IDD are miR-410 (Zhang et al., 2020), miR-31-5p (Xie et al., 2020), miR-302c (Yu et al., 2023), and the lncRNA colon adenocarcinoma hypermethylated (CAHM) (Li et al., 2022). Treatment with MSC-exosomes decreases the levels of markers of ECM degradation, such as IL-1β, cyclooxygenase (COX)-2, MMP13, and iNOS (Liang et al., 2021; Lu et al., 2021). MSC-exosomes inhibit AGE-induced ER stress in NPCs by modulating AKT and ERK signaling (Liao et al., 2019). The NLRP3 (Zhang et al., 2020; Yu et al., 2023) and mTOR/autophagy pathways are also implicated in the effects of MSC-exosomes in IDD (Liang et al., 2019). Xie *et al.* showed that MSC-exosomes protect against IDD in a rat model by inhibiting oxidative stress, an effect reversed in part by miR-31-5p knockdown (Xie et al., 2020). Li *et al.* demonstrated that MSC-exosomes inhibit M1 macrophage polarization, NPC apoptosis, ECM degradation, and IDD progression by delivering the lncRNA CAHM, an effect reversed in part by siCAHM (Li et al., 2022). Moreover, Guan *et al.* (Guan et al., 2023) reported that MSC-exosomes can modulate macrophage polarization and NPC senescence, thereby suppressing the apoptosis of NPCs and mitigating IDD (Figure 7).

### Obstacles to the use of MSC-exosomes-based therapeutics in skeletal diseases

Research has focused on the therapeutic potential of MSCs and exosomes for skeletal diseases. Besides exhibiting similar therapeutic effects, MSC-exosomes could overcome the safety and ethical concerns associated with MSCs injection. In addition, exosomes have less-stringent storage requirements than MSCs. Their efficacy and ability to be targeted to bone marrow, cartilage, and macrophages can be enhanced by bioengineering and preconditioning. These characteristics make exosomes good candidates for the treatment of skeletal diseases.

Currently, the existing clinical trials of MSC-exosomes–based therapies are mainly focused on cardiovascular disease, liver cirrhosis, psoriasis, macular holes, dry eye disease, diabetes mellitus, pneumonia, sepsis, wound healing and cancer (data from http://clinicaltrials.gov). For example, Dehghani et al. reported that no post-interventional adverse effects were observed following intraparenchymal implantation of MSC-exosomes in five ischemic stroke patients (Dehghani et al., 2022). There are also several ongoing clinical trials investigating the role of MSC-exosomes in skeletal diseases. Matas et al. are conducting an interventional clinical trial to compare the safety and efficacy of MSC-exosomes injection in patients with mild to moderate symptomatic OA (Phase I, No. NCT05060107, 2021-10-05∼2023-10-05). In another clinical trial, researchers are comparing injection of SF-MSC-exosomes with SF-MSCs in degenerative meniscal injury (Phase II, No. NCT05261360, 2022-03∼2025-03). Also, autogenous MSC culture medium that containing exosomes are being studied in a clinical trial to enhance the osteogenesis of bone grafting (Phase I/II, No. NCT04998058, 2023-12-15∼2024-12-30).

However, whether MSC-exosomes can replace MSCs, and whether stem cells and exosomes in combination have synergistic therapeutic effects, is unclear. In addition, the long-term hazards of MSC-exosome therapy are unknown, necessitating evaluation of their effect on the immune system and the bone microenvironment. Moreover, compared with the existing MSCs-related therapies, the standardized methods of isolation, characterization and purification of MSC-exosomes need to be improved in terms of their reliability, cost, yield, and reproducibility. The ability of exosomes to deliver therapeutics for skeletal diseases also merits further investigation.

## Conclusion and future directions

Exosomes have considerable therapeutic potential for skeletal diseases but are at an early stage of development. The functionality, appropriate dosage, distribution, and clearance of exosomes need to be investigated to ensure their safety and efficacy. Their clinical application is impeded by a variety of challenges; therefore, further research is needed.
